# Cardiac Arrest Simulation Response Time: A Comparative Study in Preclinical Medical Education

**DOI:** 10.7759/cureus.81572

**Published:** 2025-04-01

**Authors:** Mari Stefani Ascano-Ravelo, Danielle L Drew, Elias M Bouyounes, Tom Lindsey, Michael J Parks

**Affiliations:** 1 Center for Simulation and Technology, VCOM-Carolinas, Spartanburg, USA

**Keywords:** cardiac arrhythmia, emergency medicine resuscitation, medical student training, response time, simulation in medical education, skills and simulation training

## Abstract

Introduction

Medical students often face a critical challenge when transitioning from academia to real-world clinical practice, especially in life-or-death situations such as cardiac arrests. Despite extensive classroom training, newly trained physicians often feel unprepared for actual emergencies. Simulation-based learning has emerged as a crucial tool in bridging this gap, offering students realistic scenarios for practicing life-saving skills, such as cardiopulmonary resuscitation (CPR). This study aimed to assess whether increased exposure to simulated cardiac arrest scenarios enhances the response time of first-year medical students when initiating CPR.

Methods

A total of 29 first-year medical students were randomized into two groups: the "lethal treatment arm" and the "normal treatment arm," differing in the frequency of CPR simulations. All participants completed a pre-test, followed by six practice simulations, and a final test. The time to initiate CPR ("time to chest") was recorded and compared between the two groups.

Results

Results showed significant reductions in CPR response times from the initial to final simulation for both groups, though no statistically significant difference was found between the two groups in terms of response time improvement.

Conclusion

This suggests that even limited exposure to CPR simulations significantly enhances medical students' readiness to respond to cardiac arrests. Further research is needed to determine the optimal quantity and frequency of simulation training for maintaining high-quality CPR performance among medical students.

## Introduction

When faced with life-or-death situations, individuals often confront a critical juncture: will they freeze in fear or will their training and instincts guide them through the crisis? This dilemma is particularly substantial for medical students, whose initial experiences in real clinical environments can be profoundly impactful and even traumatic. Despite their preparation to lead in life-saving emergencies, newly minted physicians often feel ill-equipped for actual clinical practice [1]. Therefore, it becomes increasingly important to evaluate the readiness of medical students as they transition from theoretical learning to hands-on application.

In the fast-paced realm of healthcare, the transition from textbook expertise to clinical knowledge marks a pivotal milestone. One such skill that requires repetitive practice and in-depth knowledge is performing and leading a cardiopulmonary resuscitation (CPR) attempt. Advanced resuscitation measures take on a whole new meaning when a life is on the line and the hypothetical clock is ticking, where seconds can mean the difference between life and death. Moments such as these can only be taught in the gripping face of reality and the tangible effects of experience that no amount of textbook study can fully capture.

The comprehensive medical curriculum spans four rigorous years, with the first two years dedicated to understanding the human body. However, translating classroom learning into adept, hands-on skills during these first two years remains a challenge. Acknowledging this gap, many medical institutions have increasingly embraced simulation as an indispensable educational tool. Simulation technologies, ranging from immersive virtual reality (VR) platforms to interactive patient scenarios, provide medical students with an authentic preview of the complex challenges awaiting them in clinical practice. High-fidelity manikins, equipped to mimic vital signs and respond dynamically to interventions such as CPR and chest tube insertions, provide a safe yet realistic environment for honing life-saving skills [2].

The use of simulation in medical schools mimics its foundational uses in hospitals for training and developing necessary skills among healthcare professionals. Hospitals employ simulation-based training to sharpen responses to critical scenarios such as cardiac arrests, where timely intervention profoundly impacts patient outcomes [3]. Simulation training has been used to improve pediatric and emergency medicine residents in their pediatric resuscitation [4]. The use of simulation within the emergency department setting has provided an effective way to improve diagnostic reasoning among medical students who lack clinical exposure [5]. Despite the recognized importance of simulation-based teaching in enhancing emergency response times, limited quantitative studies assess its success in cardiac arrest scenarios among medical students.

For instance, a study by Langdorf et al. underscored that even seasoned fourth-year medical students took nearly two minutes to initiate CPR in simulated cardiac arrest scenarios, emphasizing the pivotal role of experience in refining reaction times [6]. Building on this foundation, the present study seeks to quantify the influence of increased exposure to cardiac arrest simulations on the response rate of first-year medical students. Our hypothesis suggests that students immersed in higher-frequency simulation scenarios will demonstrate quicker initiation of CPR compared to their counterparts with fewer exposure opportunities.

In conclusion, the integration of simulation-based learning early in medical education not only bolsters technical proficiency but also fosters the rapid, assured decision-making crucial in healthcare settings. As the landscape of medical education evolves, bridging the chasm between classroom instruction and real-world needs remains paramount in cultivating future healthcare leaders capable of navigating life-and-death situations with competence and compassion. By embracing immersive simulation technologies, medical schools can better prepare their students to confront the daunting challenges of clinical practice, ensuring that each new generation of healthcare providers stands ready to deliver timely and effective care when it matters most.

This article was previously presented as a poster presentation at the 2024 VCOM Research Day on February 9, 2024, and the 2024 American College of Osteopathic Internists (ACOI) Annual Convention and Scientific Sessions on November 1, 2024.

## Materials and methods

A comparative study involving 29 first-year medical students was conducted at the simulation center on the Carolinas campus of the Edward Via College of Osteopathic Medicine (VCOM) in Spartanburg, SC, USA, to assess response times during simulated scenarios based on prior exposure to cardiac arrest scenarios. IRB approval was obtained from the VCOM Institutional Review Board (IRB #2023-135) prior to initiating this study to ensure compliance with ethical standards and the protection of participants' rights. The study was conducted over a span of seven weeks. To recruit potential participants, an email was sent out to the medical school’s first-year cohort by the investigators to initiate the recruitment process, containing the recruitment flyer. Investigators used a one-week time period for recruitment. At the end of the one week, the research team began the informed consent process with each individual, consisting of face-to-face meetings or via email, in which the study's purpose, procedures, expected duration, risks, benefits, confidentiality measures, and any other relevant information were discussed. Potential participants had at least a week to address questions and concerns and complete the consent form. After consents were completed and prior to participating, participants completed a demographic pre-survey detailing any prior exposure to cardiac arrest situations, such as having ever witnessed or responded to a cardiac arrest. With the exception of their response to prior cardiac arrest experiences, the demographic characteristics of the participants were not recorded. Participants were emailed to sign up for a time slot for their first simulation. Prior to starting the first simulation, individuals were provided information on how to take the pulse of the high-fidelity manikin, the use of a telemetry screen, and the location of the code cart.

The simulations were conducted individually. Each participant was asked to go into the simulation bay and begin the scenario as if it were a real-life event inside a hospital setting. Each participant was given an asystole (flat-line on monitor) scenario where the manikin transitioned into a pulseless and unresponsive state. The event had a maximum time of 10 minutes for each of the participants. All initial simulations were timed and video-recorded. The key metric of interest, "time to chest," was defined as the interval between the onset of cardiac arrest (asystole) and the initiation of hands-on cardiac resuscitation (CPR) during both their initial and final simulated scenarios.

Following the initial simulations, the participants were divided into two treatment groups using a balanced stratified randomization process based on their previous cardiac arrest experience. To standardize their understanding, all participants received a comprehensive PowerPoint presentation on basic electrocardiograms (EKGs) prior to commencing practice sessions. Subsequently, each participant engaged in a series of six practice scenarios, with one group, dubbed “lethal treatment arm,” receiving more simulations requiring CPR than the other group, dubbed “normal treatment arm,” before their final scenario. The simulations requiring CPR consisted of a randomized amount of ventricular tachycardia and ventricular fibrillation. The lethal treatment arm received five lethal heart rhythms (requiring CPR) and one normal sinus rhythm (not requiring CPR) and the normal treatment arm received two lethal heart rhythms and four normal sinus rhythms. These practice sessions were not recorded. Participants were encouraged to refer to their EKG presentation to aid with these practice sessions. Each practice simulation lasted no longer than 10 minutes to ensure consistency and efficiency throughout the study. Once all participants finished all six practice sessions, each participant underwent one final simulation. The final simulation was identical to the initial simulation and was timed and video-recorded. All simulations, both recorded and practice, were completed within a four-week timeframe. After data collection, the spreadsheet was provided to a faculty member not participating in the research to key code the data to de-identify the participants. Once the data had been de-identified, the data analysis began.

The study originally involved 32 first-year medical students; however, due to a deviation in the intended simulation delivery for two participants during the practice sessions, their data was excluded from the final analysis. Statistical analyses were performed using SAS 9.4 software (SAS Institute Inc., Cary, NC) with a two-tailed type I error rate set at 0.05. Subjects were assigned randomly to treatment groups using a permuted block design to ensure balanced allocation. Fisher’s exact test was used to examine whether randomization had equally distributed baseline characteristics, such as previous experience as a first responder or training.

Data evaluation through histograms and boxplots identified outliers in the pre-intervention response times for both groups, as well as outliers in the change of response times within each group. To account for these outliers and the small sample size, the Wilcoxon signed rank test was utilized to assess significant reductions in response times within each group. Furthermore, the Wilcoxon rank sum test was used to compare the extent of decrease in response times between the treatment groups, thus evaluating the effectiveness of differing simulation exposures on response time improvements.

## Results

**Figure 1:**
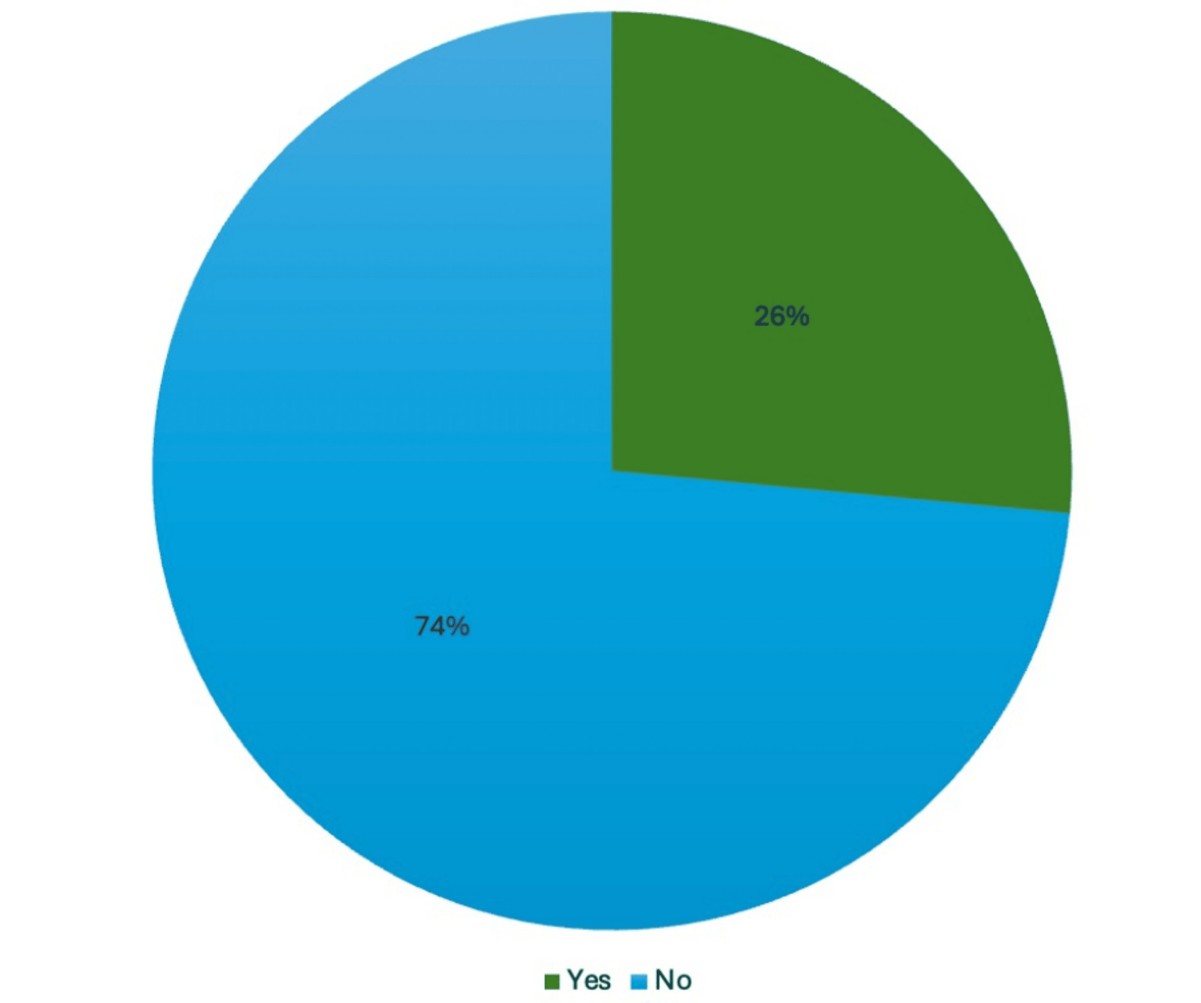
Results from the demographic pre-survey, indicating the number of participants who have had experience, or exposure at minimum, to a cardiac arrest scenario prior to medical school.

From October 2023 to November 2023, first-year medical students were recruited for this study via email from the investigators. There were no exclusion criteria; however, students were screened based on their experience in witnessing and/or responding to cardiac arrest scenarios. No other demographic characteristics were recorded. Among the 32 recruited students, 7 had prior experience, while 25 had no prior experience (Figure 1).

**Figure 2:**
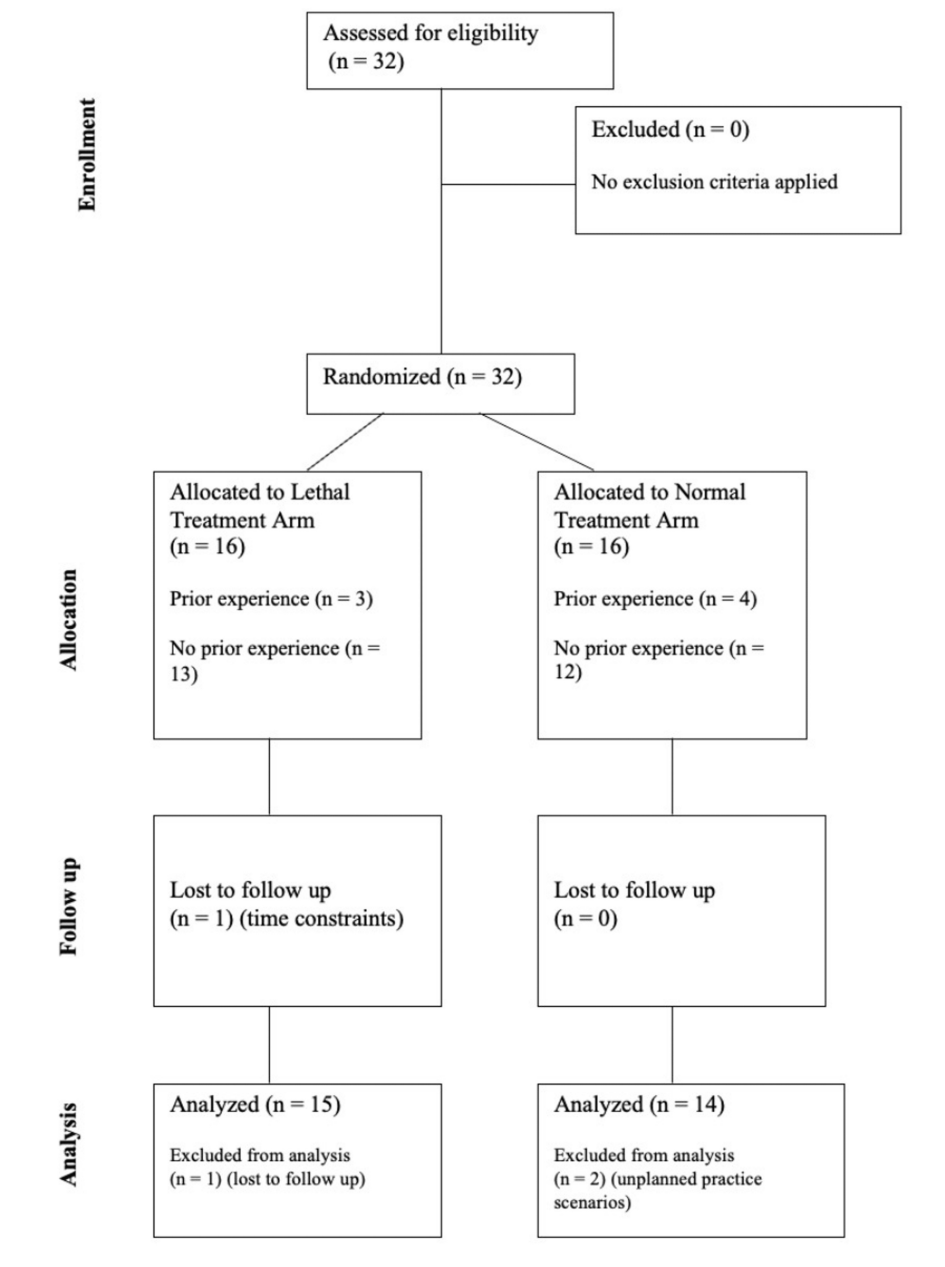
CONSORT diagram of participant flow performed in this randomized, comparative study. CONSORT, Consolidated Standards of Reporting Trials

Participants were given approximately five weeks, from November 2024 to December 2024, to complete the initial simulation, six practice simulations, and a final simulation. Students who could not complete their practice scenarios during the scheduled times in the simulation bays were offered alternative dates with the research team. One student was lost to follow-up during the first half of practice simulations, and two students received unplanned scenarios during their practice. Consequently, data from a total of three participants were excluded from the data analysis. The recruitment, randomization, follow-up, and analysis of the participants can be seen in the CONSORT flow diagram in Figure 2.

**Figure 3:**
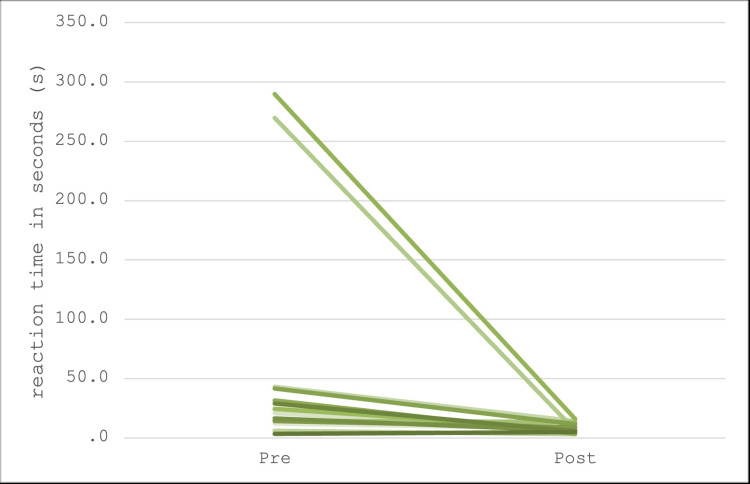
The lethal treatment arm had a median decrease in response times from pre- to post-intervention of 15.5 seconds.

**Figure 4:**
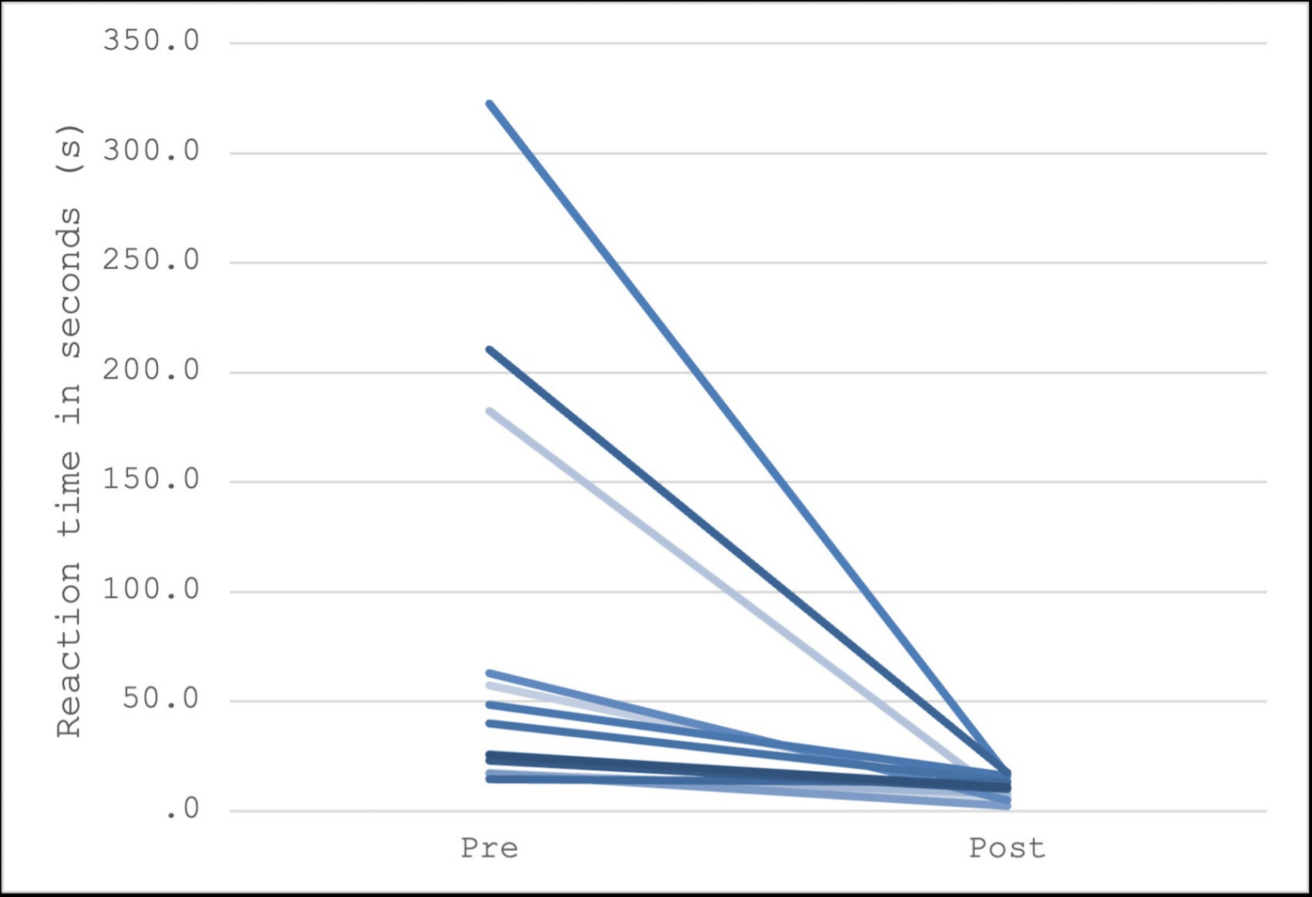
The normal treatment arm had a median decrease in response times from pre- to post-intervention of 21.25 seconds.

Upon completion of the initial simulations, the 32 participants were divided into groups of two, with 16 students in each group. Group assignments were randomized, but the distribution of students with prior experience was balanced across both groups. Fisher’s exact test was employed to verify the equal randomization of each group. The testing procedures were identical for both groups. For the participants assigned to the lethal scenario, referred to as the "lethal treatment arm," a median decrease in response times of 15.5 seconds was observed from pre- to post-intervention. This group exhibited a minimum decrease of -1.5 seconds and a maximum decrease of 274 seconds, as seen in the individual line plot in Figure 3. Analysis revealed a statistically significant median decrease (p < 0.001) using the Wilcoxon signed rank test, accounting for the larger outliers. Participants in the "normal treatment arm" demonstrated a median decrease in response times of 21.25 seconds from pre- to post-intervention. This group exhibited a minimum decrease of 1.5 seconds and a maximum decrease of 306 seconds, as seen in the individual line plot in Figure 4. Similar to the lethal treatment arm, this group also showed a statistically significant median decrease (p < 0.001) according to the Wilcoxon signed rank test.

However, when comparing the median decrease of 15.5 seconds in the lethal treatment arm to the 21.25 seconds in the normal treatment arm, analysis using the Wilcoxon rank sum test did not achieve statistical significance (p = 0.2750); therefore, the statement that the observed median decreases for the two groups are equal cannot be rejected.

## Discussion

Upon completion of the initial simulations, the 32 participants were divided into two groups of 16, with balanced prior experience across groups. The lethal treatment arm showed a median decrease in response times of 15.5 seconds from pre- to post-intervention, with a range from -1.5 seconds to 274 seconds. The normal treatment arm had a median decrease of 21.25 seconds, ranging from 1.5 seconds to 306 seconds. Both groups demonstrated a statistically significant reduction in response time (p < 0.001), as confirmed by the Wilcoxon signed rank test. However, a comparison of the median decreases between groups using the Wilcoxon rank sum test (p = 0.2750) did not reach statistical significance, meaning we cannot reject the statement that both groups had equivalent reductions in response time.

Although not all physicians are faced with daily life-or-death situations, every physician must be prepared if such an event were to occur under their watch. All medical students are trained in identifying clinical emergencies and responding to pulseless patients with CPR [6]. This vital skill is not simply learned from a textbook or lecture. It requires guidance from an experienced provider and practice [7]. Recent graduates point to a lack of direct clinical experience in medical school as the largest deficiency in their training [1]. In order to better prepare medical students for the real-life scenarios they would inevitably encounter during their clinical rotations, medical schools have adopted simulation equipment meant to provide near realistic patient care experiences. Although these tools could never replace clinical experience, they give students a chance to safely learn lifesaving skills. Simulation has begun to integrate VR and even artificial intelligence to enhance students’ learning experiences through interactive, immersive environments [8]. McCoy et al. showed that high-fidelity manikin simulation can significantly improve CPR performance by fourth-year medical students, which included a more rapid EMS activation [2]. However, not much research has been done delving into the quantity of simulation necessary to improve medical students’ clinical skills, especially in their preclinical years. Simulation-based training has positive impacts on the medical professional’s response time to a critical scenario, specifically cardiac resuscitation [9]. Does the quantity of simulation training change a student’s proficiency in responding to a cardiac arrest with CPR? Based on our results, the students with an increased amount of cardiac arrest scenarios did not statistically differ from the students with less scenarios. This is contrary to our hypothesis that there would be a significant difference in time to begin CPR between the two groups. Both groups were equal in reducing their response to a cardiac arrest from their initial to final scenario. However, all students showed a statistically significant reduction in time taken to begin CPR from their initial to final scenario. The data suggest that students do not need repetitive CPR training within one month to improve their response time. Instead, one to two practice simulations may be sufficient to significantly reduce the student’s response time in a real-life cardiac arrest scenario.

This research is limited by the small sample size of 29 participants, which would potentially reduce the generalizability of our data. There is also the possibility of cross-contamination due to unintentional communication between participants in different groups since the simulations were conducted over multiple days. However, these limitations do not invalidate the result that simulation training does reduce a medical student’s CPR response time, regardless of the simulation quantity. Simulation-based resuscitation training can also enhance healthcare professionals' confidence in executing CPR [10]. We recommend further research to determine if the quantity of simulation training would improve the quality of CPR conducted by medical students [2]. Furthermore, research should be conducted to determine the frequency of CPR training in order to maintain proficient response times and high-quality CPR among not only medical students but also residents and practicing physicians [11].

## Conclusions

In our randomized, comparative study of first-year medical students, we found that students in the lethal treatment arm had no statistically significant difference in “time to chest” compared to students in the normal treatment arm when comparing their times from initial to final simulation scenario. All students showed a significant reduction in “time to chest” from initial to final scenario, therefore reinforcing the benefits of simulation training in medical education. Medical students are given the opportunity to improve their skills in a safe and controlled environment to better prepare themselves for the real life-and-death scenarios they will face during their clinical training and future practice. Further research should be conducted to explore the effect of simulation quantity on CPR quality and simulation frequency on the maintenance of CPR response proficiency.
